# Bibliometric analysis of chondrocyte apoptosis in knee osteoarthritis

**DOI:** 10.1097/MD.0000000000040000

**Published:** 2024-10-04

**Authors:** Hongxing Zhang, Yao Yang, Minglei Gao, Jiafeng Peng, Danyang Li, Junchen Zhu

**Affiliations:** a Department of Second Clinical Medical College, Anhui University of Chinese Medicine, Hefei, Anhui Province, China; b Department of Orthopaedics, Second Affiliated Hospital of Anhui University of Chinese Medicine, Hefei, Anhui Province, China.

**Keywords:** bibliometric, cell apoptosis, knee arthritis, visualization analysis

## Abstract

**Background:**

Apoptosis, a form of programmed cell death, plays a significant role in osteoarthritis; however, bibliometric studies in this field remain scarce. Bibliometrics provides a visual representation of research outcomes and trends, guiding future investigations.

**Method:**

Journal data from January 1, 2013, to December 31, 2023, in this field were obtained from the Web of Science (WOS) core database. Analysis was conducted using VOSviewer and CiteSpace.

**Results:**

Analysis revealed that over the past decade, 794 articles were published in 299 journals by 4447 authors from 49 countries and 877 institutions. The top contributors were China, the United States, and the United Kingdom. Zhuang Chao emerged as the most prolific author, and “osteoarthritis and cartilage” ranked as the most frequently cited journal. Keyword clustering focused on mechanisms, inflammation, and cartilage. The most-cited article was “chondrocyte apoptosis in the pathogenesis of osteoarthritis” in the “International Journal of Molecular Sciences.” Burst word analysis highlighted extracellular matrix, circular RNA, micro RNA, indicating current research hotspots.

**Conclusion:**

Utilizing bibliometrics and visual analysis, we explored the hotspots and trends in the field of chondrocyte apoptosis in osteoarthritis. Extracellular matrix, Circular RNA, Micro RNA, among others, are likely to become future research focal points and frontiers.

## 1. Introduction

Osteoarthritis (OA) is a prevalent degenerative joint disease in clinical practice, causing pain and functional impairment in patients, thereby imposing substantial clinical and economic burdens.^[1]^ Osteoarthritis of the knee manifests as a series of pathological changes occurring when the regenerative capacity of the knee joint is surpassed by the damage it sustains, primarily involving alterations in articular cartilage and synovial membrane. The integrity of joints or synovial joints is crucial for connecting relevant parts of the skeleton. Typically composed of 2 articulating bony ends covered by articular cartilage, a synovial joint comprises the synovial membrane, which provides stability. Articular cartilage consists of chondrocytes within the extracellular matrix (ECM), which is produced and maintained by these chondrocytes.^[2]^ Apoptosis, also known as programmed cell death, is a tightly regulated mechanism crucial for organismal development and aging.^[3]^ For instance, following tissue injury, the accumulation of neutrophils, macrophages, and lymphocytes at the site of injury initiates tissue repair. Upon completion of the healing process, excess accumulated cells are eliminated through programmed cell death to prevent excessive inflammation and consequent tissue damage.^[4]^ Hence, dysregulation of apoptosis is associated with various diseases, including developmental defects, autoimmune disorders, and cancer. Dysregulation of microRNAs regulating oncogenes in cancer cells disrupts the balance between pro-apoptotic and antiapoptotic proteins, leading to cellular dysfunction.^[5]^ Fas and TNF-related apoptosis-inducing ligand (TRAIL) receptors, members of the tumor necrosis factor (TNF) receptor superfamily, are highly upregulated in many cancers such as hepatocellular carcinoma, ovarian cancer, and renal cell carcinoma.^[6]^ Acquired immunodeficiency syndrome results from excessive apoptosis of T cells due to internalization of human immunodeficiency virus into T cells via CD4 receptors and increased expression of Fas receptors.^[7]^ Hyun Sook Hwan et al posit a correlation between the extent of cartilage damage and chondrocyte apoptosis, suggesting chondrocyte apoptosis as an effective target for regulating cartilage degeneration. Over the years, cartilage degeneration, depletion of chondrocytes, and chondrocyte apoptosis have been central to OA research.^[8]^

Bibliometrics is a method of summarizing literature characteristics qualitatively and quantitatively to investigate the current status and trends in a specific field. It is widely applied in the medical field, aiding researchers in understanding development trends, key issues, and research frontiers across various medical topics. Khan et al conducted a bibliometric analysis of the 100 most-cited articles on cardiovascular diseases in mainland China, identifying research trends, innovations, and landmark studies in this area.^[9]^ Ke et al provided a bibliometric analysis of tumor markers in lung cancer diagnosis, summarizing the current state of research and methods to enhance the diagnostic efficiency of traditional serum tumor markers, offering new directions for improving lung cancer detection rates.^[10]^ In the field of knee osteoarthritis, a bibliometric analysis on exosome-related research identified trends and milestone studies, while also highlighting future directions in this domain.^[11]^ However, there is currently a gap in utilizing bibliometrics for the study of chondrocyte apoptosis in knee osteoarthritis. Therefore, we employed CiteSpace and VOSviewer to analyze the past 10 years of research on chondrocyte apoptosis in osteoarthritis. The analysis focused on 2 main aspects: first, an examination of the overall landscape of chondrocyte apoptosis in the OA field through the analysis of authors, countries, institutions, and journals; second, a burst analysis of keywords and references to identify research priorities and future hotspots. Through these analyses, we aim to delineate the current status and hotspots of chondrocyte apoptosis research in the OA field.

## 2. Method

### 2.1. Data sources

The literature is derived from the Web of Science (WOS), specifically the “WOS core collection,” with citation indexing set to “ALL.” The data were downloaded within 1 day on November 20, 2023. WOS, recognized as a high-quality digital literature resource, has gained widespread acceptance among researchers. This database is extensively employed for scientific metric analysis and visualization in research.^[12–14]^

### 2.2. Data retrieval

The search query “TS= (‘knee osteoarthritis’ OR ‘osteoarthritis’ OR ‘patients with osteoarthritis’ OR ‘osteoarthritis of rabbit knee’ OR ‘senile knee osteoarthritis’) AND TS= (‘chondrocyte apoptosis’)” was used in WOS to retrieve articles. The search period ranged from January 1, 2013, to December 31, 2023. Article types included were articles and review articles. A total of 887 articles and reviews were initially retrieved, and after excluding other document types, 794 articles were retained.

### 2.3. Data cleaning

After exporting literature from multiple databases, duplicate entries may occur, as some publications might be indexed in more than 1 database. To address this, reference management software should be used to remove duplicates. During the initial screening process, the titles and abstracts of the documents should be manually reviewed to exclude those that do not align with the research topic. In this study, certain papers that focused on other types of osteoarthritis, were unrelated to knee joints, or were based on animal experiments without clinical relevance, were manually excluded. To ensure the accuracy of data analysis, citation formats must be standardized, and the names of authors and institutions must be normalized to avoid double-counting research teams or institutions due to spelling inconsistencies.

**Figure 1.**
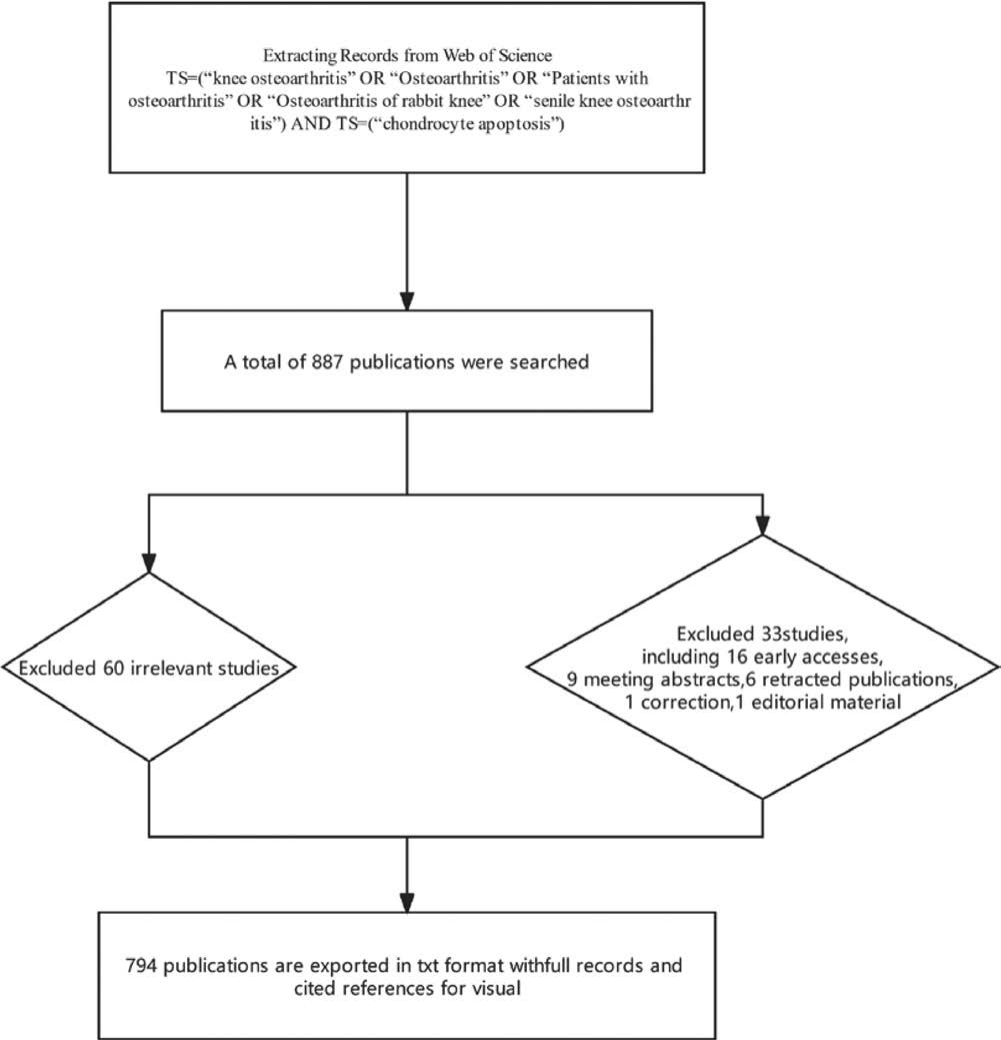
Data filtering flowchart.

A total of 887 papers were retrieved through the search terms. After the initial screening, 60 irrelevant papers were excluded. A further 33 studies that did not meet the inclusion criteria were removed, including 16 early online publications, 9 conference abstracts, 6 retracted papers, 1 correction, and 1 editorial. In the end, 794 articles were selected for analysis (Fig. 1).

### 2.4. Visualization analysis

After filtering the data in WOS, it was downloaded in plain text format and imported into CiteSpace and VOSviewer for visualization analysis. CiteSpace excels in uncovering “burst terms” and research frontiers within a field. This tool identifies keywords or topics with rapidly increasing citation frequencies, reflecting research hotspots during specific periods. Additionally, CiteSpace supports citation analysis, displaying citation bursts to track the evolution and development of research areas. In KOA research, CiteSpace can be used to analyze citation networks, identify “high-impact” papers that have sparked significant discussions, and reveal groundbreaking discoveries in the field.^[15]^ VOSviewer is a bibliometric tool known for its powerful visualization capabilities, particularly in constructing and displaying large-scale collaboration networks, keyword co-occurrence maps, and citation networks. It supports various types of visualizations, making it easier to identify core themes and emerging trends in a research field. In the study of knee osteoarthritis (KOA), VOSviewer is highly suitable for conducting keyword co-occurrence analyses and building author collaboration networks, helping researchers quickly identify key topics and leading research teams.^[16]^

### 2.5. Research ethics

The data utilized in this study do not involve patient clinical information; hence, ethical approval is not required.

## 3. Result

### 3.1. Basic quantitative information

The 794 articles utilized in this study were authored by 4447 researchers from 877 institutions spanning 49 countries. These articles were published across 299 journals and cited 24,930 articles from 2144 different journals.

### 3.2. Annual publishing trends

**Figure 2.**
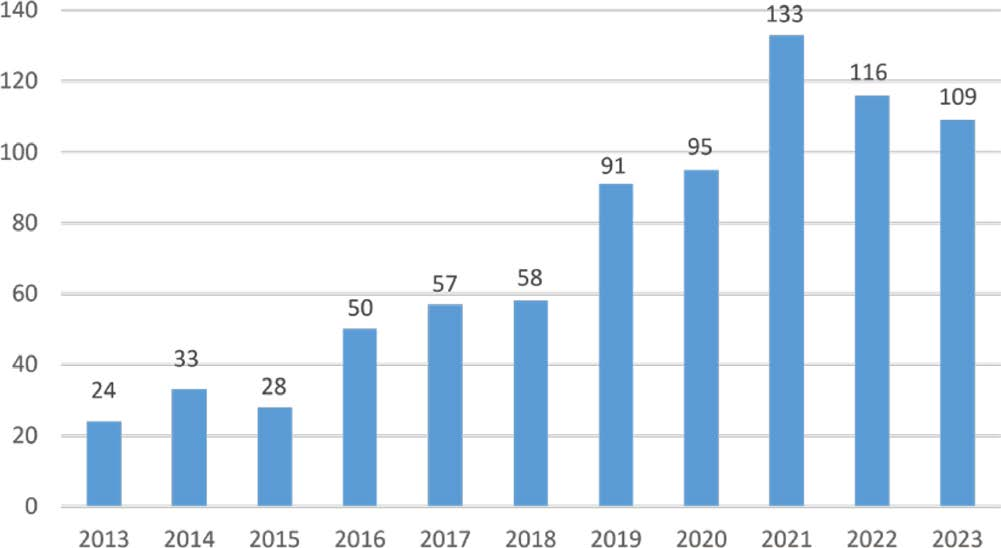
Annual publication trend chart of papers.

The annual publication count reflects the research trends in the field. Overall, there is an upward trend in research related to chondrocyte apoptosis in knee osteoarthritis. The publication count was relatively low between 2013 and 2015, indicating an early stage of research and development in this area. After 2015, there was a gradual increase in research activity, doubling approximately every 2 years. This growing interest in the study of chondrocyte apoptosis in knee osteoarthritis signifies an overall upward trend in research (Fig. 2).

### 3.3. Author, journal, country/institution information

**Figure 3.**
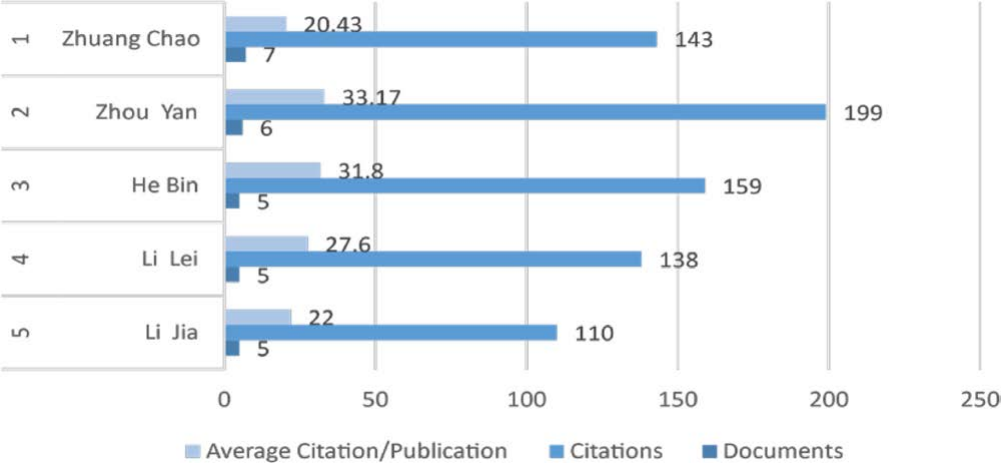
The top 5 authors in terms of publication volume.

Analyzing the literature authors allows for the identification of representative scholars in the research field. Through VOSviewer analysis and the exclusion of homonymous entities based on author affiliations, the top 5 prolific authors in terms of publication output in this field were identified. Among them, Zhuang Chao from Nanjing University of Chinese Medicine emerged as the most prolific author, having published a total of 7 papers from January 2013 to December 2023, with 143 citations and an average citation per paper of 20.43. The second-highest contributor in publication output was Zhou Yan from Wuhan University, with 6 papers, 199 citations, and an average citation per paper of approximately 33.14 (Fig. 3). Zhuang Chao and colleagues focused on the mechanisms of cell apoptosis. By isolating normal human and osteoarthritic (OA) chondrocytes, and using Q-PCR and western blot analyses to detect FSTL1 expression, they discovered that FSTL1 promotes SNP-induced chondrocyte apoptosis through the activation of the SAPK/JNK/Caspase3 signaling pathway.^[17]^ On the other hand, Zhaoyan research concentrated on the effects of small-molecule drugs on chondrocyte apoptosis. Their study found that chitosan oligosaccharides effectively inhibited IL-1β-induced chondrocyte apoptosis by activating the p38 MAPK signaling pathway, while gastrodin alleviated chondrocyte aging and mitochondrial damage in OA by regulating PI3K-AKT phosphorylation through SIRT3.^[18,19]^

Next, a statistical analysis of the journals in which the papers were published was conducted. Using VOSviewer, the top 5 journals with the highest number of publications were identified, all of which are related to the medical field. The journals with 20 or more articles include Osteoarthritis and Cartilage, Molecular Medicine Reports, and the International Journal of Molecular Sciences, with 25, 21, and 20 articles, respectively. Notably, Osteoarthritis and Cartilage falls under the Q1 category in the JCR rankings, specifically in the fields of orthopedics and rheumatology. One of its published papers, “CAMKK2 is upregulated in primary human osteoarthritis and its inhibition protects against chondrocyte apoptosis,” discusses how calcium/calmodulin-dependent protein kinase kinase 2 (CAMKK2) is upregulated in OA cartilage, correlating with increased levels of pro-apoptotic and catabolic proteins. Inhibition or knockdown of CAMKK2 reduces chondrocyte apoptosis and catabolic protein levels, while its overexpression increases them. CAMKK2 may serve as a potential therapeutic target for preventing or alleviating human OA, offering a new direction for knee osteoarthritis treatment.^[20]^

**Figure 4.**
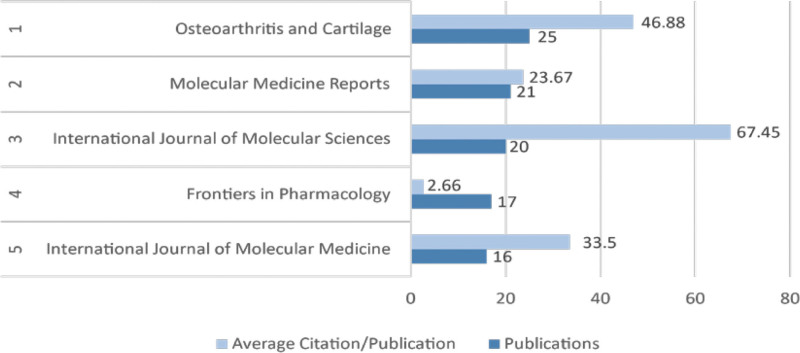
The top 5 journals in terms of publication volume.

The journal with the highest average citations per article is the International Journal of Molecular Sciences, with 20 papers and an average of 60.75 citations per article, indicating the high-quality of its publications and the significant attention they have received in the field (Fig. 4).

**Figure 5.**
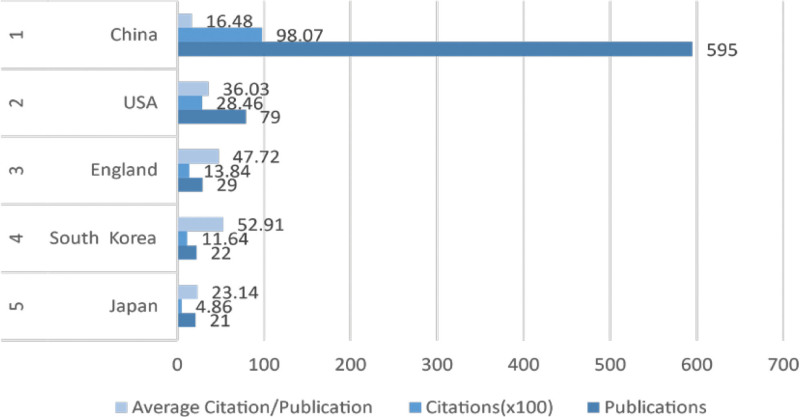
The top 5 countries in terms of publication volume.

Further analysis of the high-productivity countries in this field, as revealed by VOSviewer, indicates that the top 5 countries in terms of productivity are, in descending order, China, the United States, the United Kingdom, South Korea, and Japan. Among these, Chinese scholars have the highest publication output (595 articles), garnering 9807 citations, albeit with a relatively lower average citation per paper. Following China, the United States has published 79 articles with 2846 citations. South Korea leads in the average citation per paper category, with 22 articles receiving 1164 citations, averaging an impressive 52.91 citations per paper (Fig. 5).

To identify the leading contributors in the field, we conducted a VOSviewer analysis on the publication output of 49 countries. For visualization, countries with a publication count equal to or >3 were selected. The results are illustrated in the figure, where larger circles indicate higher publication output. The connecting lines between nodes represent the strength of collaboration, with thicker lines indicating more frequent joint publications between 2 countries. Node colors represent different clusters. The graphical representation underscores a considerable imbalance in the distribution of publishing countries in this field, with a prominent “top-heavy” effect. A majority of the papers are authored by scholars from a small number of countries (Fig. 7A).

**Figure 6.**
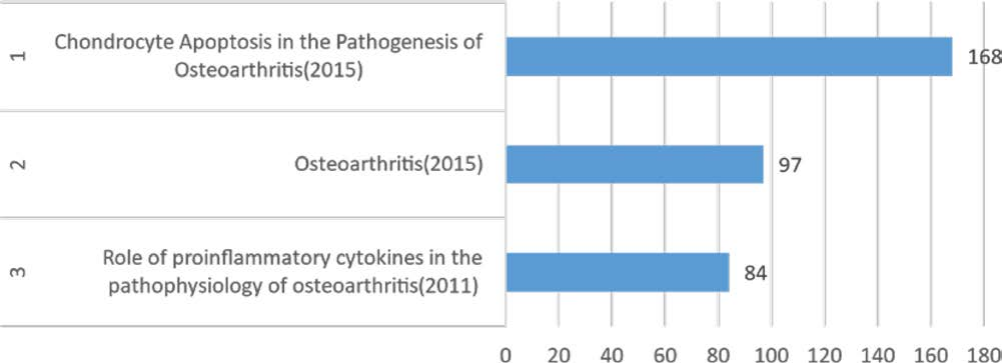
Top 3 cited literature.

### 3.4. Keyword co-occurrence analysis

**Figure 7.**
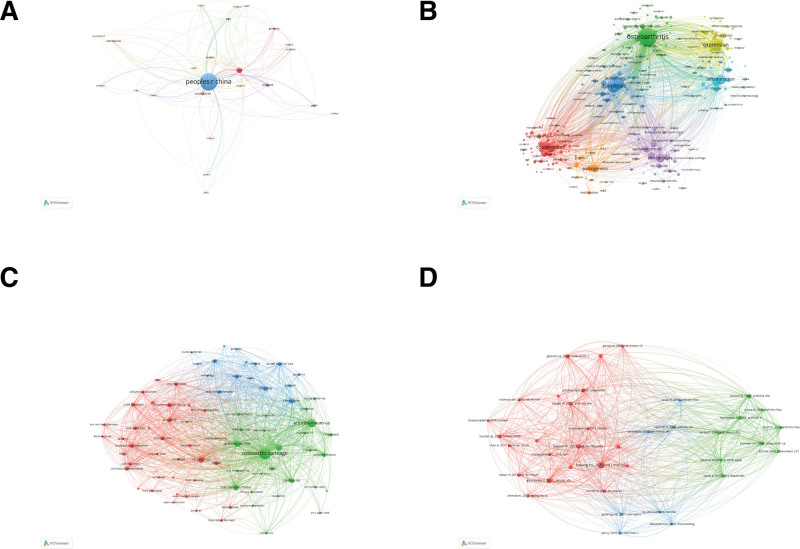
The data visualization. (A) Visualization analysis chart for countries with more than 3 publications. (B) Analysis chart of co-occurrence of keywords in the research field. (C) Co-citation map of research field journals. (D) Co-citation map of research field literature.

Keywords encapsulate the essence and core of a paper, and co-occurrence analysis of keywords can reveal research hotspots in a particular field. Using VOSviewer to analyze 894 articles, we adopted a criterion of a co-occurrence frequency of 5 times or more to include 258 keywords out of a pool of 2777. In the graph, the size of the nodes represents the frequency of keyword occurrence, reflecting the research hotspots in the field. The connecting lines between nodes indicate associations between keywords, with thicker lines denoting a higher frequency of co-appearance in the same article. Node colors signify different clusters (Fig. 7B). Keyword clusters are concentrated around mechanisms, inflammation, and cartilage.

To gain a more detailed insight into the specificities of keywords, we extracted those with frequencies exceeding 10, as illustrated in Table 1.

**Table 1:** Keywords with a frequency >10 in the research field.

Keyword	Occurrences	Total link strength
Osteoarthritis	574	2348
Apoptosis	302	1382
Expression	238	1089
Chondrocyte apoptosis	205	859
Cartilage	192	859
Inflammation	161	763
Chondrocytes	146	615
Articular cartilage	126	578
Proliferation	99	487
Autophagy	96	476

From the graph and table, it is evident that key terms such as osteoarthritis, apoptosis, expression, chondrocytes, cartilage, inflammation, proliferation, and autophagy constitute representative terminology in this field. The largest nodes in the graph are osteoarthritis and apoptosis. Research in this field, both domestically and internationally, spans molecular biology, cell biology, pathology, and clinical medicine. The etiology of knee osteoarthritis is multifaceted. Microscopically, characteristic features of OA cartilage include loss of collagen and proteoglycans, leading to disruption of the extracellular matrix structure and affecting biomechanical properties.^[21]^ Clustering of chondrocytes near the superficial layer while undergoing apoptotic death in the deep and calcified layers is observed.^[22]^ At the cellular and molecular levels, several factors contribute. Primarily, OA often involves inflammation, resulting in the release of inflammatory cytokines (e.g., TNF-α, interleukin-1).^[23,24]^ These cytokines activate cellular signaling pathways, including NF-κB (nuclear factor-κB) and MAPK (mitogen-activated protein kinase), which regulate the expression of apoptosis-related genes, thus promoting apoptosis.^[25]^ Moreover, inflammation and cellular damage may induce oxidative stress, disrupting the intracellular redox balance. Oxidative stress increases intracellular reactive oxygen species, causing oxidative damage to proteins, lipids, and nucleic acids, thereby inducing apoptosis.^[26]^ Mitochondria, the energy centers of cells, also participate in regulating apoptosis. Oxidative stress and inflammation may lead to mitochondrial dysfunction, releasing apoptotic signaling molecules such as cytochrome C, thereby activating apoptotic pathways.^[27]^ Apoptotic pathways primarily comprise 2 aspects: extrinsic (receptor-mediated) and intrinsic (mitochondrial-mediated) pathways. In knee osteoarthritis, both pathways may be activated. The extrinsic pathway typically involves death receptors, while the intrinsic pathway operates through the release of apoptotic proteins from mitochondria.^[28]^ Some antiapoptotic proteins (e.g., BCL-2 family) maintain cell survival under normal circumstances, but in knee osteoarthritis, their expression may be dysregulated, rendering cells more susceptible to apoptosis.^[29]^ During disease progression, OA chondrocytes produce matrix metalloproteinase 13 (MMP-13) capable of degrading collagen matrix, along with a disintegrin and metalloproteinase with thrombospondin motifs 5 (ADAMTS-5). Synthesis of these degrading enzymes further exacerbates cartilage breakdown.^[30]^ Biomechanical and biochemical changes collectively disrupt cartilage homeostasis and contribute to the pathogenesis of OA.^[31]^

### 3.5. Co-citation analysis

Analysis of co-citations enables an understanding of highly cited papers and the journals that publish them within the field. Utilizing VOSviewer, a co-citation network of journals was generated, setting a minimum citation threshold of 100 to retain 79 journals for co-citation analysis. The resulting co-citation network of journals is illustrated in the graph.

According to VOSviewer, the top 3 journals in terms of co-citations are “osteoarthritis and cartilage” (cited 2764 times), “arthritis and rheumatism” (cited 1420 times), and “arthritis research and therapy” (cited 839 times).

The co-citation network comprises 3 clusters. The blue cluster primarily focuses on molecular chemistry, emphasizing research on knee osteoarthritis. Journals in this cluster contribute foundational support for knee osteoarthritis research. The blue area predominantly addresses disease-related aspects of knee osteoarthritis, often originating from clinical needs, investigating problems associated with knee osteoarthritis, and providing new directions for clinical applications (Fig. 7C).

Further analysis was conducted on the co-citations of the literature. Initially, utilizing VOSviewer, the top 3 co-cited papers in the field over the past decade were identified, as presented in Figure 6.

Among these, the most-cited article is titled “chondrocyte apoptosis in the Pathogenesis of Osteoarthritis,” published in the “International Journal of Molecular Sciences,” with a total of 168 citations. This indicates the substantial impact and credibility of the article within the field. Subsequently, a co-citation network of references was visualized using VOSviewer, with a citation threshold set at 30, retaining 38 articles for co-citation analysis. The resulting co-citation network, as depicted in the figure, forms 3 distinct clusters, suggesting the establishment of foundational research in the field.

In this network, the red cluster predominantly focuses on literature review studies in the field, while the blue cluster’s articles primarily originate from the molecular chemistry domain, exploring apoptosis in chondrocytes (Fig. 7D). Additionally, a notable observation is that highly cited articles are mainly concentrated in the years 2010 to 2015, with a total of 16 articles cited 30 times or more.

### 3.6. Analysis of burst words

**Figure 8.**
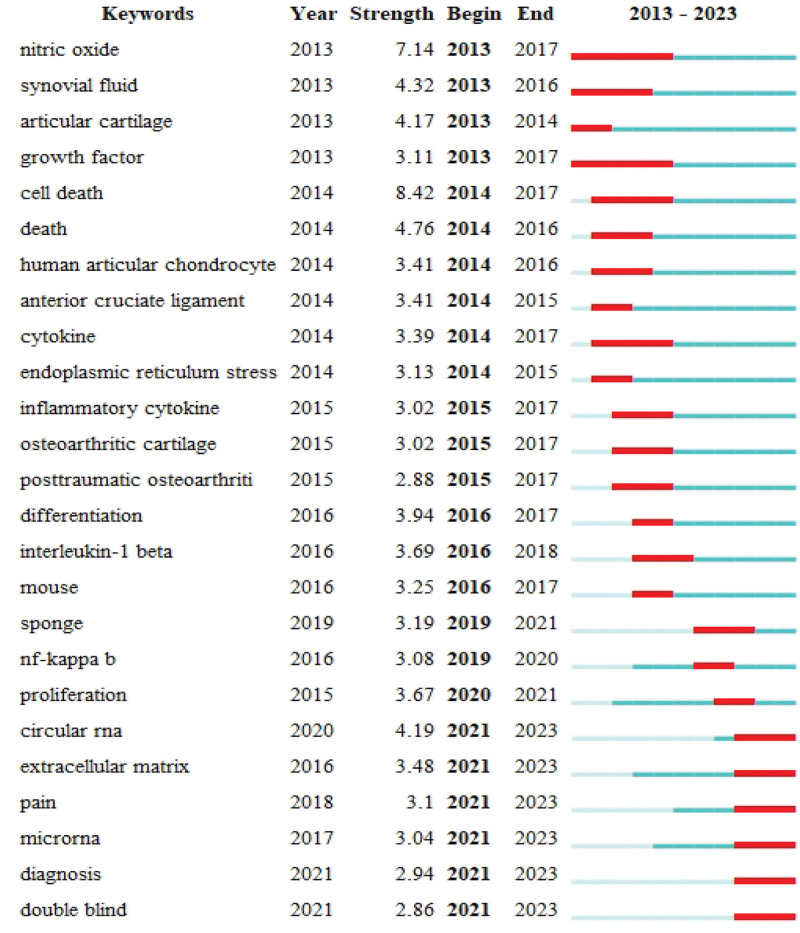
Research field burst word graph.

Burst terms refer to keywords that frequently appear within a specific time frame. Burst term analysis not only provides insights into the research hotspots during different periods in a particular field but also aids in analyzing recent research trends and predicting future directions. Utilizing CiteSpace, we obtained a burst term network for chondrocyte apoptosis, as illustrated in Figure 8. The term “nitric oxide” exhibits the highest burst strength, indicating that it was a focal point in the realm of chondrocyte apoptosis, especially before 2017. Nitric oxide can impact chondrocyte apoptosis by activating or inhibiting cell signaling pathways. Celeste M Hancock and others discovered that NO might mediate its effects by activating the cGMP signaling pathway to regulate intracellular redox reactions, alleviating knee arthritis pain.^[32]^

Additionally, NO plays a role in the inflammatory process, which is associated with chronic inflammation and cartilage diseases. The generation of nitric oxide may be linked to oxidative stress and inflammation, thereby influencing the survival and apoptosis of chondrocytes.^[33]^ NO also affects cell survival by regulating the cell cycle of chondrocytes.^[34]^ Consequently, researchers have explored therapeutic approaches targeting NO regulation, aiming to protect chondrocytes from apoptosis by modulating NO production or influencing the NO signaling pathway.^[27]^

The enduring popularity of keywords such as “extracellular matrix (ECM),” “circular RNA,” and “micro RNA” suggests that research in these areas continues to be a focal point in the current period and is likely to remain so in the foreseeable future.

Studies indicate that changes in the ECM within joint cartilage primarily manifest in alterations to collagen, proteoglycans, and glycoproteins. These changes may lead to the loss of the cartilage’s original elasticity and compressive resistance, significantly contributing to the development of knee osteoarthritis.^[35]^ There is an interplay between inflammatory reactions and ECM, where the release of inflammatory mediators influences the synthesis and degradation of ECM, thereby accelerating the breakdown of joint cartilage.^[36]^ Researchers have found that the regulation of ECM synthesis and degradation is associated with the gene expression of matrix metalloproteinases (MMPs) and tissue inhibitors of metalloproteinases (TIMPs).^[37,38]^ Moreover, abnormal biomechanical loading may contribute to the abnormal synthesis and degradation of ECM, hastening the degeneration of joint cartilage.^[39]^

Apoptosis of chondrocytes in knee osteoarthritis can also be observed through imaging techniques. Live-cell imaging using confocal microscopy can reveal the behavior of chondrocytes and their interactions with the extracellular matrix (ECM). Collagen in the cartilage provides tensile strength, while proteoglycans enhance compressive resistance which retain water. These components can be visualized through high-resolution imaging. Techniques such as high-resolution MRI with T2 mapping, T1rho imaging, and delayed gadolinium-enhanced MRI of cartilage (dGEMRIC) can assess the integrity of collagen and the proteoglycan content. Transmission electron microscopy (TEM) offers ultra-high-resolution images at the molecular level, detailing the structure of collagen fibers and proteoglycan aggregates. These high-resolution imaging techniques capture subtle changes in chondrocytes and the ECM, aiding in a comprehensive understanding of knee osteoarthritis.^[40,41]^

Both circular RNA (circRNA) and microRNA (miRNA) can modulate the expression of apoptosis-related genes in chondrocytes, influencing the survival and death of articular cartilage cells.^[42,43]^ They can also impact the level of inflammation by regulating the expression of inflammation-regulate genes.^[44,45]^ Furthermore, both can serve as potential biomarkers for diagnosing and monitoring the progression of knee osteoarthritis.^[43,46]^ Studies have found that miRNA-based therapeutic approaches, by regulating the expression of specific miRNAs, can inhibit inflammation, promote cartilage regeneration, and intervene in the development of knee osteoarthritis.^[43]^ CircRNA acts as a miRNA sponge, regulating the inhibitory effect of miRNA on target genes, thereby influencing processes such as inflammation and cell apoptosis.^[46]^

## 4. Discussion

This study utilized VOSviewer and CiteSpace software to analyze the field of knee osteoarthritis over the past decade systematically. It examined the development trends in this area and conducted analyses and discussions on highly productive authors, key journals, and keywords. The conclusions drawn are summarized as follows:

1) Through co-citation analysis, it is evident that the field has established a stable research foundation.
2) China, the United States, and the United Kingdom are the top 3 countries contributing to this field, with China and its institutions taking a leading position.
3) Influential journals in this field include “Osteoarthritis and Cartilage,” “Molecular Medicine Reports,” and “International Journal of Molecular Sciences.”
4) The paper “Chondrocyte Apoptosis in the Pathogenesis of Osteoarthritis” published in “International Journal of Molecular Sciences” has been cited 168 times, indicating high credibility and impact in the field.
Co-citation and clustering analysis of keywords revealed multiple stable research topics in this field.

Summarization of the burst words, it was found that current research hotspots mainly focus on “ ECM,” “ circRNA,” “ miRNA” among others. Burst word analysis reflects the changes in research hotspots in this field, suggesting that future research will continue to focus on these areas. In KOA research, the extracellular matrix (ECM) primarily plays a role in cartilage tissue repair and regeneration. Future studies could further explore how biomaterials, such as synthetic ECM or natural ECM derivatives, can be used to promote the regeneration and repair of joint cartilage. The regulatory role of circRNA in the pathogenesis of KOA is gaining increasing attention. Future basic research should aim to uncover the specific functions of circRNA in KOA, particularly its regulatory effects on inflammation and cartilage degeneration. Additionally, miRNA can influence KOA progression by regulating multiple gene expression networks. To apply miRNA more effectively in clinical settings, future research should focus on developing more precise and efficient miRNA delivery systems, such as nanoparticles or viral vectors, to enhance therapeutic targeting and safety.

Bibliometric studies can elucidate the current research landscape in the field and provide research directions and ideas for researchers. Its significance lies in:

1) It aids beginners in gaining a clear understanding of the field’s framework and delving into its developmental processes.
2) Analysis of burst terms assists researchers in identifying breakthroughs and hotspots in the field.
3) Journal and co-citation analyses facilitate researchers in swiftly locating relevant references and contribute to manuscript submissions in the field.

However, the study has certain limitations. Firstly, it solely relies on the WOS Core Collection database, excluding other databases, which may result in a less comprehensive analysis. Secondly, subjective biases may be introduced during quantitative analysis due to the researchers’ preexisting knowledge of the field. Future research should integrate multiple databases for a more comprehensive analysis and actively engage with scholars in the field to understand the latest developments, enhancing an objective understanding and mitigating subjectivity in the interpretation of the analysis.

## 5. Conclusion

Analysis using VOSviewer and CiteSpace software reveals a rising trend in publications on cellular apoptosis in the field of knee osteoarthritis in international journals, indicating significant academic value and potential applications. China, the United States, and the United Kingdom emerge as the top 3 contributors to this field, with China and its affiliated institutions leading the way. Multiple research themes have been established, indicating a stable research foundation. “Extracellular Matrix (ECM),” “Circular RNA,” and “Micro RNA” are current research hotspots and future research trends.

## Author contributions

**Conceptualization:** Hongxing Zhang, Junchen Zhu.

**Data curation:** Hongxing Zhang, Minglei Gao.

**Validation:** Hongxing Zhang, Jiafeng Peng.

**Writing – original draft:** Hongxing Zhang.

**Writing – review & editing:** Hongxing Zhang, Yao Yang, Danyang Li.
